# Early predictors of outcomes of hospitalization for cirrhosis and assessment of the impact of race and ethnicity at safety-net hospitals

**DOI:** 10.1371/journal.pone.0211811

**Published:** 2019-03-06

**Authors:** V. V. Pavan Kedar Mukthinuthalapati, Samuel Akinyeye, Zachary P. Fricker, Moinuddin Syed, Eric S. Orman, Lauren Nephew, Eduardo Vilar-Gomez, James Slaven, Naga Chalasani, Maya Balakrishnan, Michelle T. Long, Bashar M. Attar, Marwan Ghabril

**Affiliations:** 1 Division of Gastroenterology and Hepatology, Indiana University, Indianapolis, Indianapolis, United States of America; 2 Department of Medicine, Cook County Health and Hospitals System, Chicago, Illinois, United States of America; 3 Section of Gastroenterology and Hepatology, Baylor College of Medicine, Houston, Texas, United States of America; 4 Evans Department of Medicine, Section of Gastroenterology, Boston University School of Medicine, Boston, Massachusetts, United States of America; 5 Department of Biostatistics, Indiana University, Indianapolis, Indianapolis, United States of America; 6 Department of Gastroenterology and Hepatology, Cook County Health and Hospitals System, Chicago, Illinois, United States of America; Duke University, UNITED STATES

## Abstract

**Background:**

Safety-net hospitals provide care for racially/ethnically diverse and disadvantaged urban populations. Their hospitalized patients with cirrhosis are relatively understudied and may be vulnerable to poor outcomes and racial/ethnic disparities.

**Aims:**

To examine the outcomes of patients with cirrhosis hospitalized at regionally diverse safety-net hospitals and the impact of race/ethnicity.

**Methods:**

A study of patients with cirrhosis hospitalized at 4 safety-net hospitals in 2012 was conducted. Demographic, clinical factors, and outcomes were compared between centers and racial/ethnic groups. Study endpoints included mortality and 30-day readmission.

**Results:**

In 2012, 733 of 1,212 patients with cirrhosis were hospitalized for liver-related indications (median age 55 years, 65% male). The cohort was racially diverse (43% White, 25% black, 22% Hispanic, 3% Asian) with cirrhosis related to alcohol and viral hepatitis in 635 (87%) patients. Patients were hospitalized mainly for ascites (35%), hepatic encephalopathy (20%) and gastrointestinal bleeding (GIB) (17%). Fifty-four (7%) patients died during hospitalization and 145 (21%) survivors were readmitted within 30 days. Mortality rates ranged from 4 to 15% by center (p = .007) and from 3 to 10% by race/ethnicity (p = .03), but 30-day readmission rates were similar. Mortality was associated with Model for End-stage Liver Disease (MELD), acute-on-chronic liver failure, hepatocellular carcinoma, sodium and white blood cell count. Thirty-day readmission was associated with MELD and Charlson Comorbidity Index >4, with lower risk for GIB. We did not observe geographic or racial/ethnic differences in hospital outcomes in the risk-adjusted analysis.

**Conclusions:**

Hospital mortality and 30-day readmission in patients with cirrhosis at safety-net hospitals are associated with disease severity and comorbidities, with lower readmissions in patients admitted for GIB. Despite geographic and racial/ethnic differences in hospital mortality, these factors were not independently associated with mortality.

## Introduction

Hospitalized patients with cirrhosis represent a significant burden to health systems, with high mortality, prolonged stays and early readmission rates. Hospitalization costs account for more than 50% of the economic burden of care for patients with end stage liver disease [1]. Prior studies on outcomes of hospitalization in cirrhosis and the predictors of key metrics, including mortality and 30-day readmissions, describe predominantly liver transplant center experiences. In contrast to transplant centers, safety-net hospitals have the legal mandate to care for all populations. Thus, unlike non-safety net hospitals, they typically serve predominantly disadvantaged urban populations with limited access to care and high rates of Medicaid or no medical insurance [2, 3]. Longitudinal data suggest that hospital performance for care of myocardial infarction and pneumonia is lower for safety-net hospitals with higher mortality and early readmissions, and smaller gains in performance over time [3, 4]. Disparities in clinical processes of care appear to be driven by racial/ethnic, socioeconomic, and geographic considerations for safety-net hospitals and the populations they serve [5–7]. In addition, differences in quality of care provided to patients with cirrhosis by hepatologists from the same academic institutions can vary based on the health system setting, with lower rates of variceal screening and liver transplant access at safety-net hospitals [8]. Therefore patients with cirrhosis admitted to safety net hospitals may be particularly vulnerable to poor outcomes, potentially worse outcomes of hospitalization, and may be impacted by racial/ethnic disparities, but are relatively understudied.

The “Cirrhosis in Urban Safety-net Hospitals” (CrUSH) study is a regionally and racially diverse multicenter study of urban safety-net hospitals. It was designed to examine hospitalization and long-term outcomes of hospitalized patients with cirrhosis. The study data were analyzed to: 1) characterize hospitalized patients with cirrhosis and their outcomes, 2) describe the early predictors of hospital mortality, 30-day readmission and prolonged length of stay, and 3) assess the association of race/ethnicity with hospital mortality and 30-day readmission.

## Methods

### Study design and participants

This is a multicenter retrospective cohort study that includes four safety net hospitals: Ben Taub General Hospital (Baylor College of Medicine, Houston, Texas), Boston Medical Center (Boston University, Boston, Massachusetts), Eskenazi Health Hospital (Indiana University School of Medicine, Indianapolis, IN), and John Stroger Hospital (Cook County Health, Chicago, IL). This retrospective and minimal risk study was approved by the Institutional Review Board at each study center, and informed consent was waived. Patients with cirrhosis admitted to the hospital for any reason between January 1 2012 to December 31 2013 were identified in each center’s administrative databases using International Classification of Disease 9 codes for cirrhosis and related complications as previously described [9, 10] Table A in S1 File). These administrative codes were still in use during the study period, (predating the adoption of ICD 10 codes), with planned follow up for at least 2 years. All identified hospital admissions in adults with cirrhosis were reviewed and cirrhosis was confirmed by radiographic changes of cirrhosis and portal hypertensive complications and/or histology. The reasons for hospitalization, and their hierarchy in cases of multiple indications, were determined from the clinical impressions in the course of care based on documentation including admission and discharge notes.

For the purposes of the present study, patients admitted in 2012 for at least one liver related indication were included. Liver related indications (based on the documented clinical impressions in the course of care) were categorized as ascites, hepatic encephalopathy, gastrointestinal bleeding (GIB) related to portal hypertension, fluid overload (edema or anasarca), alcoholic hepatitis (in patients with cirrhosis), spontaneous bacterial peritonitis, liver malignancy, hepatic hydrothorax, acute kidney injury, and jaundice. Hospitalizations for surgical care or in liver transplant recipients were excluded. In patients with multiple admissions, the first admission during the study period was analyzed. Clinical data including liver related complication and mortality were identified on chart review and not using administrative codes. Patients were followed after discharge until the last available follow-up or liver transplantation (LT) in survivors or death in non-survivors.

### Statistical analysis

Data collected included demographic and insurance information, the reason for hospitalization per clinical documentation, disposition on admission, the etiology and prior complications of cirrhosis, and the course and outcomes of hospitalization. Liver disease severity was assessed using the Child Pugh Score (CPS) and Model for End-stage Liver Disease (MELD), and medical comorbidities were assessed using the Charlson Comorbidity Index (CCI) [11]. The North American Consortium for the Study of End-stage Liver Disease-Acute on Chronic Liver Failure (NACSELD-ACLF) definition was used to categorize patients with acute on chronic liver failure on the first day of hospitalization [12]. A series of teleconferences throughout the study were used to maximize homogeneity in methodology and approach to data collection using the same Access database (Microsoft, Redmond, Washington), and de-identified data was collated at Indiana University. The collated data was fully anonymized.

Patient and hospitalization characteristics were compared among the four study centers using descriptive analysis. The main outcomes were hospital mortality, all cause same-center 30-day readmissions, and length of stay. Continuous variables were described as median values (interquartile range) and categorical variables as number (percentage). Comparisons of continuous variables between groups was analyzed by the Mann-Whitney test or Analysis of Variance test. Comparisons of categorical variables was analyzed using the Chi-square test. Simple logistic regression was used to identify the clinical factors on the first day of hospitalization that were independently associated with hospital mortality, 30-day readmission, and prolonged length of stay (defined as the highest decile). Clinical predictors were internally validated with bootstrapping analysis [13, 14]. The bootstrapping analysis was performed by sampling with replacement from the population, creating 10,000 bootstrapped samples of the same size as the original sample, in order to determine which variables remained significantly associated with the outcomes [15]. Due to some variables having some missing data, multiple imputation was performed, as well, with the bootstrapping method, and compared to the initial models, where data with missing values were dropped from the analyses [16]. The final multiple logistic regression models were informed by post-estimation analysis using Akaike Information Criteria [17] and the area under the receiver operator characteristic curve (reported as c-statistic). The performance of the final models predicting hospital mortality, 30-day readmission and prolonged length of stay were tested in each center’s cohort using c-statistic. All analyses were performed using SAS v9.4 (SAS Institute, Cary, NC) and Stata version 15 (Statacorp, College Station, TX), with significance set at a p value<0.05.

## Results

### Baseline demographic and clinical characteristics

A total of 1,212 patients with cirrhosis were hospitalized during the calendar year 2012 at the four study centers comprising 1% of hospitalized patients at all centers in that year. We excluded 100 patients admitted for surgical care, 378 patients admitted for medical care but without liver related indications, and 1 patient with a prior liver transplant. The study cohort comprised 733 patients admitted with at least one liver related indication, including 182 at Ben Taub Hospital, 173 at Boston Medical Center, 142 at Eskenazi Health Hospital, and 236 at John Stroger Hospital (**Table B in** S1 File). The Ben Taub Hospital cohort was younger with a larger proportion of Hispanic patients and lower CCI compared with the other three centers (Table 1). Most patients were admitted via the emergency department. The most common liver related indications for hospitalization were ascites, hepatic encephalopathy, and GIB. The most common etiologies of liver disease were alcohol and viral hepatitis in 635 patients (87%), although Ben Taub Hospital had a notably higher proportion of patients with fatty and cryptogenic liver disease (21% vs. 4% to 9% at other centers) (Table 2). The median MELD was 16 (IQR 12–21) with similar MELD, CPS and Child Pugh across centers. Less than half of all patients reported complete abstinence from alcohol, while 39% reported daily alcohol use (the majority >2 drinks/day), 37% used tobacco, and 16% reported active substance abuse.

**Table 1:** Demographics, insurance, selected comorbidities, primary liver related diagnosis and admission disposition in the study cohort and in 733 patients hospitalized at the respective study centers. Data are reported as number (percent) or median value (interquartile range).

Clinical factor (number of patients if there is missing data)	All patients N = 733	Ben Taub Hospital n = 182	Boston Medical Center n = 173	Eskenazi Health Hospital n = 142	John Stroger Hospital n = 236
*Age	55 (49–61)	52 (48–58)	56 (50–63)	54 (49–58)	57 (50–63)
**Gender male**	497 (68)	113 (62)	117 (68)	99 (70)	168 (71)
*Race (729) **White** **Black** **Hispanic** **Asian** **Other**	313 (43) 182 (25) 164 (22) 23 (3) 43 (6)	54 (30) 18 (10) 102 (56) 5 (3) 3 (1.7)	74 (43) 63 (36) 26 (15) 5 (3) 5 (3)	94 (66) 35 (25) 9 (6) 1 (1) 3 (2)	91 (40) 66 (29) 27 (12) 12 (5) 34 (15)
*Charlson Comorbidity Index	3 (3–5)	3 (1–4)	4 (3–5)	4 (3–5)	3 (3–5)
Comorbidities *Diabetes mellitus *Diabetes mellitus with complications *Congestive heart failure *Chronic obstructive pulmonary disease **Acquired immunodeficiency syndrome**	156 (21) 33 (5) 56 (8) 66 (9) 15 (2)	53 (29) 2 (1) 7 (4) 6 (3.3) 2 (1)	48 (28) 12 (7) 19 (11) 34 (20) 4 (2)	34 (24) 10 (7) 11 (8) 20 (14) 2 (1)	44 (19) 9 (4) 19 (8) 6 (2.5) 7 (3)
*Medical insurance **(n = 725)** **Medicaid** **Medicare** **Commercial** **County** **Jail** **No insurance** **Other**	317 (44) 114 (16) 35 (5) 114 (16) 9 (1) 121 (17) 15 (2)	46 (52) 12 (7) 8 (4) 82 (45) 2 (1) 27 (15) None	90 (52) 53 (31) 20 (12) None 2 (1) 6 (4) None	60 (42) 33 (23) 5 (4) 27 (19) 3 (2) 14 (10) None	121 (51) 16 (7) 2 (1) 5 (2) 2 (1) 74 (31) 15 (6)
**Intensive care on admission**	106 (15)	16 (9)	34 (20)	24 (17)	32 (14)
*Primary liver related diagnosis **Ascites** **Hepatic encephalopathy** **Gastrointestinal bleeding** **Alcoholic hepatitis** **Acute kidney injury** **Fluid overload** **Spontaneous bacterial peritonitis** **Liver malignancy** **Hepatohydrothorax** **Jaundice**	253 (35) 144 (20) 121 (17) 54 (7) 41 (6) 39 (5) 32 (4) 32 (4) 9 (1) 8 (1)	79 (43) 42 (23) 47 (26) 2 (1) 1 (0.5) 1 (0.5) 7 (4) None 2 (1) 1 (0.5)	36 (21) 32 (18) 23 (13) 20 (12) 5 (3) 29 (17) 10 (6) 10 (6) 5 (3) 3 (2)	34 (24) 46 (32) 31 (22) 13 (9) 2 (1) 3 (2) 8 (6) 3 (2) 1 (0.7) 1 (0.7)	104 (44) 24 (10) 20 (8) 19 (8) 33 (14) 6 (2.5) 7 (3) 19 (8) 1 (0.5) 3 (1)
*Infection on admission	147 (20)	33 (18)	33 (19)	45 (32)	36 (15)

* p value <0.05 for comparisons.

**Table 2:** Etiology and severity of liver disease on admission in the study cohort and in patients hospitalized at the respective study centers. Data are reported as number (percent) or median value (interquartile range).

Clinical factor (number of patients if there is missing data)	All patients N = 733	Ben Taub Hospital n = 182	Boston Medical Center n = 173	Eskenazi Health Hospital n = 142	John Stroger Hospital n = 236
Etiology of liver disease * Alcohol * **Alcohol and viral** Viral *Fatty liver *Cryptogenic	303 (41) 207 (28) 125 (17) 29 (4) 44 (6)	81 (45) 34 (19) 28 (15) 14 (8) 24 (13)	49 (28) 74 (43) 33 (19) 5 (3) 10 (6)	57 (40) 48 (34) 26 (18) 3 (2) 3 (2)	116 (49) 51 (22) 38 (16) 7 (3) 7 (3)
**Child Pugh score (n = 602)	8 (7–10)	9 (7–10)	8 (7–11)	8 (7–10)	9 (7–11)
*¥**Child Pugh class** **(n = 612)** **A** **B** **C**	88 (14) 310 (65) 214 (35)	14 (9) 89 (56) 56 (35)	30 (19) 66 (43) 59 (38)	24 (18) 71 (54) 36 (27)	20 (12) 84 (50) 63 (38)
**NACSELD-ACLF**	24 (3)	4 (2)	9 (5)	7 (5)	4 (2)
**Model for End-stage Liver Disease (n = 633)**	16 (12–21)	16 (12–22)	15 (12–20)	15 (12–22)	16 (13–22)
**INR (n = 651)**	1.5 (1.3–1.8)	1.5 (1.3–1.8)	1.4 (1.2–1.7)	1.4 (1.2–1.7)	1.5 (1.3–1.9)
**Creatinine (mg/dL) (n = 730)**	0.9 (0.7–1.4)	0.9 (0.7–1.3)	0.8 (0.7–1.4)	0.9 (0.7–1.3)	0.9 (0.6–1.4)
**Bilirubin (mg/dL) (n = 708)**	2.3 (1.2–4.7)	1.9 (1.2–4.4)	2.4 (1.2–4.6)	2.6 (1.6–5.9)	2.4 (1.2–4.8)
*Albumin (g/L) (n = 714)	2.7 (2.3–3.1)	2.5 (2.1–2.8)	2.8 (2.4–3.3)	2.9 (2.5–3.3)	2.6 (2.2–3.2)
*Na (mEq/L) (n = 730)	136 (132–139)	137 (134–140)	136 (133–139)	136 (133–139)	134 (130–138)
**White blood cell count (per microL)**	7 (5–10)	7 (5–11)	6 (4–9)	7 (5–10)	7 (5–9)
*Mean arterial pressure (mmHg) (n = 710)	91 (81–102)	92 (79–102)	94 (84–106)	91 (83–100)	88 (80–99)
*Ascites **(n = 694)** **None** **Controlled** **Uncontrolled**	368 (53) 180 (26) 146 (21)	103 (57) 37 (20) 42 (23)	48 (36) 48 (36) 38 (28)	91 (64) 35 (25) 16 (11)	122 (52) 62 (26) 52 (22)
*Hepatic encephalopathy **(n = 674)** **None** **Controlled** **Uncontrolled**	518 (77) 86 (13) 70 (10)	149 (82) 22 (12) 11 (6)	52 (46) 24 (21) 38 (33)	114 (80) 22(15) 6 (4)	203 (86) 18 (8) 15 (6)
*Bleeding esophageal/gastric varices	89 (12)	34 (19)	21 (12)	11 (8)	23 (10)
*Hepatocellular carcinoma	49 (7)	6 (3)	19 (11)	9 (6)	15 (6)
*Spontaneous bacterial peritonitis	23 (3)	5 (3)	10 (6)	6 (4)	2 (1)
*Number of home medications (n = 676)	5 (2–9)	4 (1–8)	10 (5–14)	5 (2–8)	3 (1–6)
*Evidence of non-compliance	90 (12)	7 (4)	25 (14)	31 (22)	27 (11)
*Active substance abuse	120 (16)	59 (32)	23 (13)	19 (13)	19 (8)
*Tobacco use	269 (37)	38 (21)	86 (50)	70 (49)	75 (32)
**Alcohol use **(n = 725)** **None** **<1 per week** **<1 per day** **1–2 per day** **>2 per day** **Unknown**	321 (44) 40 (6) 28 (4) 33 (5) 245 (34) 57 (8)	87 (48) 11 (6) 3 (2) 5 (3) 58 (32) 16 (9)	72 (43) 5 (3) 7 (4) 4 (2) 65 (39) 16 (9)	56 (40) 7 (5) 8 (6) 12 (9) 42 (34) 16 (11)	106 (45) 17 (7) 10 (4) 12 (5) 80 (34) 10 (4)

Abbreviation: INR, International Normalized Ratio, NACSELD-ACLF, North American Consortium for the Study of End-stage Liver Disease-Acute on Chronic Liver Failure

* p value <0.05 for comparisons,

** p value = .05 to.09 for comparisons.

^¥^ Includes 10 subjects with a Child Pugh Score>9 despite some missing component data

### Study outcomes and associated predictors

In total 323 patients (44%) had more than one liver related diagnosis treated during hospitalization (Table 3.) The median length of stay was 4 days (IQR 2–7) and was similar in the four centers. The in-hospital mortality rate was 7% (n = 54). Compared with hospital survivors, patients who died during the hospitalization had more frequent infection, NACSLED-ACLF, hepatocellular carcinoma (HCC) and requirement for intensive care at admission, and had higher MELD and Child Pugh scores, but lower serum sodium levels on admission (**Table C in** S1 File). There were no differences in use of non-selective beta blockers or prophylactic antibiotics on admission. The hospital mortality rates for the five most frequent liver related indications were: 11.1% for alcoholic hepatitis, 9.8% for AKI, 9.7% for hepatic encephalopathy, 9.1% for GIB and 5.1% for ascites. The clinical factors independently associated with hospital mortality included MELD, NACLSED-ACLF, HCC, sodium and white blood cell count (Table 4). Patients with HCC were more likely to have limited code status on admission (16% vs. 2%, p < .001) and through hospitalization (20% vs. 8%, p < .001) compared with patients without HCC.

**Table 3:** The liver related diagnoses associated with the hospitalization in the study cohort, and outcomes of hospitalization, including mortality and early readmission. Data are reported as number (percent) or median value (interquartile range).

Clinical factor (number of patients if there is missing data)	All patients N = 733	Ben Taub Hospital n = 182	Boston Medical Center n = 173	Eskenazi Health Hospital n = 142	John Stroger Hospital n = 236
Liver related diagnosis/conditions treated *Ascites *Hepatic encephalopathy *Gastrointestinal bleeding *Acute kidney injury *Fluid overload *Alcoholic hepatitis *Liver malignancy **Spontaneous bacterial peritonitis** Hepatohydrothorax *Jaundice	349 (48) 194 (26) 136 (19) 171 (23) 103 (14) 61 (12) 51 (7) 43 (6) 20 (3) 27 (4)	99 (54) 47 (26) 52 (29) 46 (25) 39 (21) 11 (6) None 12 (7) 2 (1) 16 (9)	57 (57) 52 (30) 25 (14) 21 (12) 42 (24) 29 (17) 18 (10) 12 (7) 5 (3) 5 (3)	58 (41) 82 (42) 37 (26) 24 (17) 11 (8) 21 (15) 8 (6) 10 (7) 7 (5) 2 (1)	135 (57) 36 (15) 22 (9) 80 (34) 11 (5) 47 (20) 25 (11) 9 (4) 6 (2.5) 4 (2)
**Received intensive care**	167(23)	34 (19)	48 (28)	32 (23)	53 (23)
**Length of stay (days)**	4 (2–7)	4 (3–7)	3 (2–6)	4 (2–8)	4 (3–7)
*In-hospital mortality	54 (7)	8 (4)	15 (9)	19 (13)	12 (5)
***Data in 679 patients discharged alive***					
*Disposition on discharge **(n = 677)** **Home or assisted living** **Skilled or long-term care** **Palliative care** **Left against medical advice** **Other (jail, other hospital)**	N = 677 558 (82) 62 (9) 17 (2.5) 19 (3) 21 (3)	n = 173 158 (87) 2 (1) 3 (2) 3 (2) 7 (4)	n = 157 111 (65) 35 (20) 2 (1) 8 (5) 1 (0.5)	n = 123 100 (70) 15 (11) 1 (1) 4 (3) 3 (2)	n = 224 189 (80) 10 (4) 11 (5) 4 (1.7) 10 (4)
**30-day all-cause readmission	145 (21)	25 (14)	38 (24)	26 (21)	56 (25)
**30-day liver related readmission**	88 (13)	20 11 ()	20 (13)	17 (14)	31 (14)
*Followed up in GI clinic	282 (42%)	74 (43%)	91 (57%)	44 (36%)	73 (33%)
**Interval to GI clinic follow up (days)**	57 (23–173)	98 (51–177)	52 (22–162)	149 (37–274)	36 (18–68)
*30-day mortality (n = 572) *90-day mortality (n = 511)	19 (3) 48 (9)	3 (2) 9 (7)	2 (1.4) 6 (4)	3 (2.6) 7 (7)	11 (7) 26 (19)

* p value <0.05 for comparisons.

** p value = .05 to.09 for comparisons.

**Table 4:** The early predictors of hospital mortality on simple and multiple logistic regression.

	Simple logistic regression		Multiple logistic regression	
Clinical variable	Odds Ratio (95%CI)	P value	Odds Ratio (95%CI)	P value
**Model for End-stage Liver Disease**	1.14 (1.1–1.19)	< .001	1.11 (1.07–1.16)	< .001
**NACSELD-ACLF**	23.4 (9.8–56)	< .001	22.5 (7.6–66.6)	< .001
**Hepatocellular carcinoma**	2.7 (1.2–6.1)	.02	5 (1.9–12.9)	.001
**Serum sodium (mEq /dL)**	0.9 (0.86–0.95)	< .001	0.92 (0.87–0.98)	.005
**White blood cell count (per microL)**	1.06 (1.01–1.1)	.01	1.03 (1.01–1.05)	.013
**Infection on admission**	3.6 (2–6.4)	< .001		
**Intensive care on admission**	4.5 (2.5–8.1)	< .001		
**Race** **(relative to White patients)** **Black** **Hispanic**	0.8 (0.4–1.6) 0.29 (.11–0.75)	.5 .01		
**Mean arterial pressure (mmHg)**	0.96 (0.94–0.98)	< .001		
**Child Pugh class C**	4.8 (2.6–9.1)	< .001		
**Study Center** **(relative to Ben Taub General Hospital)** **Boston University Medical Center** **Eskenazi Health** **John Stroger Hospital**	2 (0.9–5) 3.4 (1.4–7.9) 1.2 (0.5–2.9)	.11 .006 .7		

Abbreviation: NACSELD-ACLF, North American Consortium for the Study of End-stage Liver Disease-Acute on Chronic Liver Failure

Factors not associated with hospital mortality included: age, sex, race, etiology of liver disease, Charlson Comorbidity index, medical insurance (commercial or Medicare vs. other (Medicaid, uninsured, county, and jail related insurance)), non-selective beta blocker at the time of admission, liver related indication for hospitalization.

The final model was informed by post-estimation analysis with a c-statistic of 0.88 (95% CI 0.84–0.93).

There were 145 (21%) all-cause 30-day readmissions amongst the 679 discharged patients. The reasons for early readmission were liver-related in 89 cases (13% of all discharges) and were predominantly for ascites and hepatic encephalopathy (**Table D in** S1 File). Patients with early readmission had higher CCI, Child Pugh class, and number of medications at discharge and were less frequently initially hospitalized for GIB (**Table E in** S1 File). All-cause 30-day readmission was associated with significantly higher 30-day (odds ratio 3.1 (95%CI 1.2–7.8), p = .02) and 90-day (odds ratio 3.8 (95%CI 2–6.9), p < .001) mortality. There was no difference in 90-day mortality in patients with liver-related (19%) and non-liver related (22%) 30-day readmission (p = 0.7). MELD and a high comorbidity burden (CCI > 4 based on sensitivity analysis) were independently associated with increased risk of 30-day readmissions, whereas hospitalization for GIB was associated with lower risk of early readmission (Table 5). The predictors of liver-related 30-day readmission on multiple logistic regression analysis included MELD (OR: 1.05, (95%CI 1.01–1.09), p = .005) and high comorbidity burden (OR: 1.8 (95% CI 1.1–3), p = .03). A competing risk analysis for 30-day readmission with death as a competing risk yielded similar results.

**Table 5:** The predictors of all-cause 30-day readmission on simple and multiple logistic regression.

	Simple logistic regression		Multiple logistic regression	
Clinical variable	Odds Ratio (95%CI)	P value	Odds Ratio (95%CI)	P value
**MELD**	1.06 (1.03–1.1)	< .001	1.05 (1.02–1.09)	.001
**Primary liver related diagnosis** **Gastrointestinal bleeding (portal hypertension)**	0.21 (0.1–0.47)	< .001	0.27 (0.11–0.64)	.003
*Charlson Comorbidity Index>4	2.5 (1.7–3.7)	< .001	1.8 (1.14–2.8)	.01
**Number of medications on discharge**	1.1 (1.02–1.11)	.001		
**Child Pugh class C**	1.7 (1.1–2.6)	.02		
**Study Center** **(relative to Ben Taub General Hospital)** **Boston University Medical Center** **Eskenazi Health** **John Stroger Hospital**	1.8 (1.1–2.9) 1.6 (0.9–2.9) 2 (1.2–3.3)	.03 .13 .01		

*Multiple comorbidities were associated with early readmission and were represented by the Charlson Comorbidity Index in this analysis.

Factors not associated with 30-day readmission included: age, sex, race, etiology of liver disease, medical insurance (commercial or Medicare vs. other (Medicaid, uninsured, county, jail)), and laboratory values (admission INR, bilirubin, white blood cell count, or sodium, and last INR, bilirubin, or sodium) discharge disposition, and follow up in GI clinic.

The final model was informed by post-estimation analysis with a c-statistic of 0.68 (95% CI 0.63–0.73).

Patients who died during hospitalization had longer median length of stay (7 days (IQR 4–16)) compared with survivors (4 days (IQR 2–7)), p < .001. The threshold for the highest decile of length of stay as > 10 days (median 13 days (IQR 11–18)). Hospitalization survivors with prolonged length of stay had higher MELD, CCI, and had more frequent ACLF, infection and intensive care on admission (**Table F in** S1 File). These factors, with the exception of CCI, were independently associated with prolonged length of stay (**Table G in** S1 File).

Finally, the performance of the models predicting hospital mortality, 30-day readmission and prolonged length of stay were assessed in each study center’s cohort (**Table H in** S1 File). The models predicting mortality and prolonged length of stay performed consistently better than the model predicting 30-day readmission. Results from bootstrapped multiple imputation models were similar to the non-imputed results for all the models described (**Table I in** S1 File).

### Associations of race/ethnicity with outcomes

The study cohort was racially and ethnically diverse with differing hospital mortality rates. We compared non-Hispanic White, Black, and Hispanic patients (Table 6). Black patients were slightly older and had a higher median CCI compared to White and Hispanic patients. The higher comorbidity score in blacks was largely related to increased atherosclerotic disease and malignancies (**Table J in** S1 File). Black patients also had more frequent viral etiology of liver disease, HCC, and higher MELD (driven by worse renal function on admission). Hispanic patients had more frequent diabetes, fatty and cryptogenic liver disease, and hospitalization for GIB. We did not observe an increased mortality rate in black patients relative to White patients, and the lower hospital mortality risk for Hispanic patients compared to white patients dissipated in the risk-adjusted analysis (Table 4).

**Table 6:** Comparison of selected characteristics and outcomes of hospitalization in 659 White, Black, and Hispanic patients (23 Asian and 43 patients of other race were not included in this comparison due to the small numbers represented). Data are reported as number (percent) or median value (interquartile range).

Clinical factor (number of patients if there is missing data)	White (n = 312)	Black (n = 182)	Hispanic (n = 164)	P value
**Age**	54 (49–58)	56 (52–62)	54 (47–61)	.002
**Gender male**	218 (70)	120 (66)	109 (66)	.6
**Charlson Comorbidity Index**	3 (3–4)	4 (3–5)	3 (3–4)	< .001
**Diabetes mellitus (any)**	67 (21)	46 (25)	34 (34)	.01
**Study center** **Boston Medical Center** **Ben Taub Hospital** **Eskenazi Health Hospital** **John Stroger Hospital**	74 (24) 54 (17) 94 (30) 91 (29)	63 (35) 18 (10) 35 (19) 66 (36)	26 (16) 102 (62) 9 (5) 27 (17)	< .001
**Medical Insurance** **(n = 651)** **Medicaid** **Medicare** **Commercial** **County** **Jail** **No insurance** **Other**	143 (46) 46 (15) 17 (5) 40 (13) 5 (1.6) 54 (17) 6 (2)	80 (44) 50 (28) 13 (7) 13 (7) 2 (1) 18 (10) 4 (2)	54 (34) 14 (9) 5 (3) 56 (35) 1 (0.6) 28 (17) 2 (1)	< .001
**Primary liver related diagnosis** **Ascites** **Hepatic encephalopathy** **Gastrointestinal bleeding** **Alcoholic hepatitis** **Acute kidney injury** **Fluid overload** **Spontaneous bacterial peritonitis** **Liver malignancy** **Hepatohydrothorax** **Jaundice**	115 (37) 62 (20) 55 (18) 27 (9) 11 (4) 13 (4) 18 (6) 5 (1.6) 4 (1.2) 3 (1)	53 (29) 36 (20) 16 (9) 18 (10) 20 (11) 14 (8) 6 (3) 15 (8) 2 (1) 2 (1)	55 (35) 36 (22) 41 (25) 4 (2) 4 (2) 8 (5) 7 (4) 3 (2) 3 (2) 3 (2)	< .001
**Infection on admission**	69 (22)	44 (24)	29 (18)	.3
**Etiology of liver disease** **Alcohol** **Alcohol and viral** **Viral** **Fatty liver** **Cryptogenic**	130 (42) 104 (33) 44 (14) 12 (4) 14 (4)	59 (32) 63 (35) 42 (23) 2 (1) 3 (1.6)	77 (47) 26 (16) 24 (15) 13 (8) 23 (14)	.02 < .001 .02 .006 < .001
**Child Pugh score (n = 546)**	9 (7–10)	8 (7–11)	8 (7–10)	.4
**Child Pugh class** **(n = 555)** **A** **B** **C**	40 (15) 134 (49) 100 (36)	25 (17) 66 (45) 57 (38)	18 (14) 75 (56) 40 (30)	.4
**NACSLED-ACLF**	13 (4)	5 (3)	3 (2)	.4
**Model for End-stage Liver Disease (n = 567)**	16 (12–21)	17 (13–23)	15 (12–19)	.02
**e**	1.5 (1.3–1.8)	1.4 (1.2–1.8)	1.5 (1.3–1.8)	.8
**Creatinine (mg/dL) (n = 656)**	0.8 (0.6–1.2)	1.1 (0.8–1.8)	0.9 (0.7–1.3)	< .001
**Bilirubin (mg/dL) (n = 638)**	2.5 (1.4–5.5)	2.2 (1–4.9)	1.8 (1.1–3.2)	.003
**Albumin (g/L) (n = 642)**	2.7 (2.4–3.2)	2.7 (2.1–3.2)	2.6 (2.3–3)	.09
**Na (mEq/L) (n = 656)**	135 (132–139)	136 (132–139)	136 (133–140)	.02
**Mean arterial pressure (mmHg) (n = 453)**	90 (80–100)	96 (88–112)	92 (79–102)	< .001
**Ascites (n = 624)**	157 (53)	78 (46)	65 (41)	.04
**Hepatic encephalopathy (n = 604)**	75 (26)	35 (21)	36 (23)	.4
**Bleeding esophageal/gastric varices**	40 (13)	9 (5)	34 (21)	< .001
**Hepatocellular carcinoma**	14 (4)	21 (12)	9 (5)	.008
**Number of home medications (n = 607)**	5 (2–9)	6 (2–11)	5 (2–8)	.03
**Evidence of non-compliance**	41 (13)	22 (12)	16 (10)	.6
**Substance abuse**	50 (16)	27 (15)	34 (21)	.3
**Smoking**	142 (45)	74 (41)	31 (19)	< .001
**Alcohol use** **(n = 469)** **None** **<1 per week** **<1 per day** **1–2 per day** **>2 per day** **Unknown**	123 (40) 16 (5) 15 (5) 14 (5) 115 (37) 24 (8)	74 (41) 13 (7) 7 (4) 14 (8) 58 (32) 12 (8)	92 (56) 7 (4) 2 (1) 3 (2) 46 (28) 13 (8)	.03
**Intensive care on admission (n = 645)**	50 (16)	25 (14)	18 (11)	.3
**Length of stay (days)**	4 (2–7)	5 (3–8)	4 (3–6)	.8
**Hospital mortality**	31 (10)	15 (8)	5 (3)	.03
**30-day readmission (n = 433)**	63 (22)	37 (22)	29 (18)	.6
**30-day mortality (n = 429)** **90-day mortality (n = 40)**	6 (2.5) 18 (8)	9 (6) 16 (12)	3 (2) 11 (9)	.09 .5

Abbreviation: INR, International Normalized Ratio, NACSELD-ACLF, North American Consortium for the Study of End-stage Liver Disease-Acute on Chronic Liver Failure

### Long- term outcomes

At the time of index hospitalization, 22 (3.2%) patients were undergoing LT evaluation, 2 (0.3%) were listed, 204 (30%) had evident contraindications for LT, 366 (53.9%) were not referred, and LT referral status was unknown in 85 (12.5%). Over a median follow up of 9 months (IQR, 52 days to 2.8 years), 8 (1.2%) patients underwent LT at intervals ranging from 5 to 19 months (including 6 (1.9%) of 312 patients with MELD≥ 15 on admission) and 118 (17.4%) died. In all, 420 patients had 1,322 subsequent hospitalizations (mean 2±3, median 1 (IQR, 0 to 3) per patient). Transplant free survival rates were 83% at 1 year and 76% at 3 years. The observed 1 and 3 year transplant free survival rates were lower in black (80% and 71%) and Hispanic (80% and 71%) patients relative to White patients (86% and 79%), (p = .14 for both comparisons). On post-hoc multivariate Cox Proportional Hazard Regression analysis, MELD on index admission, CCI>4 and HCC were the predictors of decreased transplant free survival (**Table K in** S1 File).

## Discussion

This is a large, regionally diverse multicenter study of patients with cirrhosis hospitalized at urban safety-net hospitals. The study included a well-characterized and racially/ethnically diverse cohort and examines the outcomes of patients with cirrhosis admitted at safety net hospitals, an understudied and largely ignored population. We found that hospital mortality was 7% and 30-day readmission rate was 21%.

We developed and internally validated models to predict key metrics of hospitalization based on data available on the first day of admission, with models performing well across study centers. Similar to the NACSELD cohort we observed a strong association of ACLF and white blood cell count with hospital mortality, even though ACLF was diagnosed in only 3% of our cohort on admission [18]. The hospital mortality risk associated with HCC was unexpected, and may have been related to advanced malignancy and limited code status in this subgroup [19] and/or limited access to HCC treatment and liver transplantation at safety-net hospitals [20]. The main predictors of mortality also predicted prolonged length of stay, itself an important endpoint that is not commonly analyzed.

Contrasting our study cohort with hospitalized patients with cirrhosis from predominantly non-safety-net hospital and transplant center based studies, we noted greater racial diversity (57% vs. 20–40% non-whites) and slightly lower MELD (median 16 vs. 18–19), but similar rates of complications of liver disease, including HCC [21–24]. Hospital mortality in patients with cirrhosis has improved over the previous decade to 5.4% in 2010 and 7.7% in 2013 in the National Inpatient Sample and Veterans Administration, respectively [25, 26]. We observed a similar hospital mortality rate of 7% in our 2012 cohort, suggesting that our safety-net hospitals did not have a higher rate of hospital mortality.

The 30-day readmission rate (21%) for our cohort was in the reported range of 10–50% described in a recent systematic review, though slightly lower than pooled estimates of 26% [27]. Our 30-day readmission model was based on early clinical predictors and performed comparatively well (c-statistic 0.68) to another reported early model based on administrative data [28]. There were notable differences in model performance between centers (c-statistic range 0.61 to 0.73), mirroring variability in all-cause 30-day readmission rates (14–25%). Interestingly liver-related readmission rates were more homogenous (11–14%). These data highlight the challenges in predicting 30-day readmission, and the role of factors and conditions beyond the severity of liver disease in determining risk for early readmissions. Thirty-day readmission was associated with increased 90-day mortality regardless of the reason for rehospitalization (liver or non-liver related), supporting the importance of all-cause early readmission as a meaningful outcome in patients with cirrhosis.

An essential aspect of study design was the quantification of comorbidity using the CCI, which is surprisingly uncommon in similar studies. Medical comorbidities were recorded on chart review rather than using administrative codes. Not surprisingly a high comorbidity burden (CCI>4) was associated with 30-day readmission and negatively impacted transplant-free survival. The lower rate of early readmission after hospitalization for GIB in our cohort was novel, but not surprising given the reported trend toward improved outcomes of variceal bleeding in general [29]. The study cohort lacked data on primary care follow up post-discharge and only captured specialty follow up when provided at the study centers. Less than half of patients had gastroenterology clinic follow up, and less than 8% of patients who followed up did so within 2 weeks of discharge, although this was not associated with lower readmission rates. The reasons for this are likely complex, but these figures highlight the need to study and optimize transition of care and access to outpatient care for patients with cirrhosis at our centers.

Few of the differences between study centers could be explained by their geographic location, with a notably larger Hispanic population at Ben Taub General Hospital in Houston, which had the highest rates of fatty and cryptogenic liver disease. Regardless, the severity of liver disease by MELD and CPS was similar amongst centers. Alcohol and viral hepatitis were by far the main drivers of cirrhosis across all centers, with a high rate of ongoing alcohol use. These data emphasize the potential downstream benefits of systematic attention to screening and treatment of alcohol abuse [30], and addressing reported gaps in hepatitis C screening and access to direct acting antiviral therapy at safety-net hospitals [7, 31].

We expected to see racial disparities in outcomes with worse outcomes for black and Hispanic patients [32], but noted similar mortality in black relative to white patients, while unadjusted hospital mortality was lower in Hispanic patients. The very fact that study patients received cirrhosis care at these safety-net hospitals, rather than transplant centers in the same cities, may reflect common socioeconomic disadvantage among all patients, irrespective of race/ethnicity. However, we did observe black patients with cirrhosis were slightly older and suffered from more comorbidities than their White and Hispanic counterparts, including atherosclerotic conditions and malignancies (including HCC).

A striking finding in the study cohort was that very few patients underwent LT, although the reasons for this could not be discerned from the available data. These findings emphasize the need to study the potential barriers to the surveillance and treatment of HCC and access to transplant services at safety-net hospitals.

There are several limitations of this study including; i) its retrospective nature, ii) lack of data on socioeconomic factors (income, education level, primary language, and social support), iii) the administrative search may have missed some cases of cirrhosis, iv) missing data inherent to this type of study (admissions to other centers, cause of death, details on treatment provided, and some deaths after discharge) and v) we could only reliably assess same-center readmissions. The results may not be generalizable to other hospitals and health systems. The strengths of the study include the large sample size, the geographic and racial diversity and the detailed clinical characterization. The key findings were that the severity of liver disease, acuity of presentation (multi-organ failure, intensive care), and comorbidities on admission were the main determinants of mortality and 30-day readmission. Early readmissions were liver related in only 60% of cases, but were associated with higher short-term mortality regardless of indication for readmission. Despite geographic and racial/ethnic differences in hospital mortality rates, these factors were not independently associated with mortality. Notably we identified increased comorbid conditions, including atherosclerotic disease and malignancies in blacks patients with cirrhosis. We observed very low rates of LT relative to a heavy burden of recurrent hospitalizations and mortality over longer term follow up. While no modifiable factors were identified these data underscore the need to optimize transition of care for patients with cirrhosis, HCC surveillance and treatment, and access to transplant services at our centers. Prospective studies are needed to validate our early predictive models and to identify the interventions required to reduce poor outcomes in this patient population.

## Supporting information

S1 File Supporting tables. (DOCX)

## Abbreviations

AKI: acute kidney injury
CCI: Charlson Comorbidity Index
CPS: Child Pugh Score
GIB: gastrointestinal bleeding
HCC: hepatocellular carcinoma
IQR: interquartile range
MELD: Model for End-stage Liver Disease
NACSELD-ACLF: North American Consortium for the Study of End-stage Liver Disease-Acute on Chronic Liver Failure
