# Supplementary material for: Early predictors of outcomes of hospitalization for cirrhosis and assessment of the impact of race and ethnicity at safety-net hospitals
Source: PLoS One. 2019 Mar 6;14(3):e0211811. doi: 10.1371/journal.pone.0211811 (PMC6402644; doi:10.1371/journal.pone.0211811)
Supplement: S1 File — (DOCX) [file pone.0211811.s001.docx]

**Table A.** International Classification of Disease (ICD) 9 codes for cirrhosis and related complications used to identify patients with cirrhosis.

| **ICD-9 Codes to be used to identify patients with cirrhosis** |
| --- |
| 456.0 Esophageal varices with bleeding |
| 456.1 Esophageal varices without bleeding |
| 456.2 Varices in diseases classified elsewhere with bleeding |
| 456.21 Varices in diseases classified elsewhere without bleeding |
| 567.23 Spontaneous bacterial peritonitis (SBP) |
| 571.2 Alcoholic cirrhosis |
| 571.5 Cirrhosis without alcohol |
| 572.2 Hepatic encephalopathy |
| 572.3 Portal hypertension |
| 572.4 Hepatorenal syndrome |
| 789.59 Ascites |

**Table B.** Characteristics of study centers and hepatology services provided. All centers are non-transplant centers and provide consultation based care, with both inpatient and outpatient Gastroenterology and Hepatology services, with discharged patients following in the outpatient clinic.

| **Study center** | **Size**  **(bed number)** | **Number of patients in cohort and total number of unique hospitalized patients in 2012 (%)** | **Main teaching hospital for academic institution** | **Consulting specialist** | **Tertiary referral center for liver disease** |
| --- | --- | --- | --- | --- | --- |
| **Ben Taub General Hospital**  **(Baylor College of Medicine)** | 586 | 182 of 20,119 (0.9%) patients | Yes | Gastroenterologisits  Hepatologists | Yes |
| **Boston Medical Center**  **(Boston University)** | 567 | 173 of 20,504 patients (.84%) | Yes | Hepatologists  Transplant hepatologists | Yes |
| **Eskenazi Health Hospital**  **(Indiana University)** | 350 | 142 of 14,639 patients (.97%) | No | Gastroenterologists | No |
| **John Stroger Hospital**  **(Cook County Health)** | 450 | 236 of 18,178 patients (1.3%) | Yes | Gastroenterologists  Transplant hepatologists | Yes |

**Table C**. Comparison of selected characteristics in hospitalization survivors and non-survivors. Data are reported as number (percent) or median value (interquartile range).

| **Clinical factor (number of patients if there is missing data)** | **Hospitalization survivors**  **n=679** | **Hospitalization non-survivors**  **n=54** | **P value** |
| --- | --- | --- | --- |
| **Age** | 55 (49-61) | 53 (49-60) | .4 |
| **Gender male** | 459 (68) | 38 (70) | .4 |
| **Race**  **White**  **Black**  **Hispanic**  **Asian**  **Other** | 282 (42)  167 (25)  159 (24)  23 (3)  40 (6) | 31 (57)  15 (28)  5 (9)  None  3 (6) | .09 |
| **Charlson Comorbidity Index** | 3 (3-5) | 3 (3-5) | .7 |
| **Medical Insurance (n=725)**  **Medicaid**  **Medicare**  **Commercial**  **County**  **Jail**  **No insurance**  **Other** | 287 (43)  102(19)  31 (5)  110 (16)  8 (1)  118 (18)  15 (2) | 30 (56)  12 (22)  4 (7)  4 (7)  1 (2)  3 (6)  None | .05 |
| **Intensive care on admission (n=718)** | 85 (13) | 21 (40) | <.001 |
| **Primary liver related diagnosis**  **Ascites**  **Hepatic encephalopathy**  **Gastrointestinal bleeding**  **Alcoholic hepatitis**  **Acute kidney injury**  **Fluid overload**  **Spontaneous bacterial peritonitis**  **Liver malignancy**  **Hepatohydrothorax**  **Jaundice** | 240 (35)  130 (19)  110 (16)  48 (7)  37 (5)  36 (5)  31 (5)  31 (5)  9 (1.3)  7 (1) | 13 (24)  14 (26)  10 (24)  6 (11)  4 (7)  3 (7)  1 (2)  1 (2)  None  1 (2) | .6 |
| **Infection on admission** | 123 (18) | 24 (44) | <.001 |
| **Etiology of liver disease**  **Alcohol**  **Alcohol and viral**  **Viral**  **Fatty liver**  **Cryptogenic** | 277 (41)  190 (28)  118 (17)  29 (4)  41 (6) | 26 (48)  17 (31)  7 (13)  None  3 (6) | .3  .6  .4  .12  .9 |
| **Child Pugh score (n=602)** | 8 (7-10) | 11 (9-12) | <.001 |
| **Child Pugh class (n=612)**  **A**  **B**  **C** | 85 (15)  298 (53)  180 (32) | 3 (6)  12 (24)  34 (69) | <.001 |
| **NACSELD_ACLF** | 10 (1.5) | 14 (26) | <.001 |
| **Model for End-stage Liver Disease (n=632)** | 15 (12-20) | 27 (18-31) | <.001 |
| **INR (n=651)** | 1.5 (1.3-1.8) | 2 (1.4-2.6) | <.001 |
| **Creatinine (mg/dL) (n=730)** | 0.9 (0.7-1.3) | 1.2 (0.8-2.6) | <.001 |
| **Bilirubin (mg/dL) (n=708)** | 2.2 (1.2-4.3) | 6.6 (2.8-15.1) | <.001 |
| **Albumin (g/L) (n=714)** | 2.7 (2.3-3.2) | 2.4 (1.9-2.8) | <.001 |
| **Na (mEq/L) (n=495)** | 136 (133-139) | 133 (129-136) | <.001 |
| **White blood cell count (per microL)** | 7 (5-10) | 11 (6-17) | <.001 |
| **Mean arterial pressure (mmHg) (n=710)** | 92 (82-102) | 82 (72-92) | .002 |
| **Ascites (n=697)** | 303 (47) | 29 (58) | .13 |
| **Hepatic encephalopathy (n=674)** | 140 (22) | 16 (34) | .07 |
| **Hepatocellular carcinoma** | 41 (6) | 8 (15) | .01 |
| **Number of home medications (n=461)** | 5 (2-11) | 7 (3-9) | .6 |
| **Tobacco use** | 242 (36) | 27 (50) | .035 |
| **Length of stay (days)** | 4 (2-7) | 7 (4-16) | <.001 |

Abbreviation: INR, International Normalized Ratio, NACSELD-ACLF, North American Consortium for the Study of End-stage Liver Disease-Acute on Chronic Liver Failure

**Table D.** Primary indications for 30-day readmissions shown as number (percent).

| **Indications for 30-day readmission** | **N=145** |
| --- | --- |
| **Liver related diagnosis**  **Ascites**  **Hepatic encephalopathy**  **Gastrointestinal bleeding**  **Spontaneous bacterial peritonitis**  **Acute kidney injury**  **Alcoholic hepatitis**  **Hepatohydrothorax**  **Liver malignancy**  **Fluid overload** | 32 (22)  30 (21)  8 (6)  7 (5)  4 (3)  3 (2)  3 (2)  1 (1)  1 (1) |
| ***Other medical indications** | 54 (37 ) |
| **Surgical indications** | 3 (2 ) |

* Included 6 readmissions with additional diagnoses of acute kidney injury (2), ascites (1), hepatic encephalopathy (1), fluid overload (1) and liver malignancy (1).

**Table E**. Comparison of selected characteristics in 679 hospitalization survivors without and with all cause 30-day readmission. Data are reported as number (percent) or median value (interquartile range).

| **Clinical factor (number of patients if there is missing data)** | **No 30-day readmission**  **n=474** | **30-day readmission**  **n=145** | **P value** |
| --- | --- | --- | --- |
| **Age** | 54 (49-60) | 55 (50-62) | .6 |
| **Gender male** | 361 (68) | 98 (68) | .9 |
| **Race (n=673)**  **White**  **Black**  **Hispanic**  **Asian**  **Other** | 219 (41)  130 (25)  130 (25)  16 (3)  34 (6) | 63 (44)  37 (26)  29 (20)  7 (5)  8 (6) | .7 |
| **Charlson Comorbidity Index** | 3 (3-4) | 4 (3-5) | <.001 |
| **Diabetes mellitus** | 137 (26) | 47 (32) | .1 |
| **Medical Insurance (n=671)**  **Medicaid**  **Medicare**  **Commercial**  **County**  **Jail**  **No insurance**  **Other** | 223 (42)  70 (13)  28 (5)  92 (17)  7 (1.3)  96 (18)  10 (2) | 64 (44)  32 (22)  3 (2)  18 (12)  1 (0.7)  22 (15)  5 (3) | .05 |
| **Primary liver related diagnosis**  **Ascites**  **Hepatic encephalopathy**  **Gastrointestinal bleeding**  **Fluid overload**  **Alcoholic hepatitis**  **Spontaneous bacterial peritonitis**  **Liver malignancy**  **Hepatohydrothorax**  **Acute kidney injury**  **Jaundice** | 109 (30)  83 (23)  85 (23)  24 (7)  23 (6)  17 (5)  11 (3)  6 (2)  4 (1)  3 (1) | 32 (36)  25 (28)  6 (7)  6 (7)  7 (8)  6 (7)  6 (7)  2 (2)  3 (3)  1 (1) | .07 |
| **Infection on admission** | 90 (17) | 33 (23) | .1 |
| **Etiology of liver disease**  **Alcohol**  **Alcohol and viral**  **Viral**  **Fatty liver**  **Cryptogenic** | 217 (41)  149 (28)  95 (18)  21 (4)  33 (6) | 60 (41)  41 (28)  23 (16)  8 (6)  8 (6) | .9  .9  .6  .4  .8 |
| **Child Pugh score (n=397)** | 8 (7-10)  8.4±2 | 9 (7-11)  9.1±2.2 | .005 |
| **Child Pugh class (n=563)**  **A**  **B**  **C** | 72 (16)  248 (54)  135 (30) | 13 (12)  50 (46)  45 (42) | .05 |
| **NACSELD-ACLF** | 8 (1.5) | 2 (1.4) | .9 |
| **Model for End-stage Liver Disease (n=583)** | 15 (12-19) | 18 (13-23) | <.001 |
| **INR (n=601)** | 1.4 (1.3-1.7) | 1.4 (1.3-1.9) | .7 |
| **Creatinine (mg/dL) (n=676)** | 0.9 (0.7-1.2) | 1.1 (0.7-1.8) | <.001 |
| **Bilirubin (mg/dL) (n=656)** | 2.1 (1.1-4.1) | 2.5 (1.3-4.8) | .17 |
| **Albumin (g/L) (n=662)** | 2.7 (2.3-3.2) | 2.7 (2.4-3.1) | .6 |
| **Na (mEq/L) (n=453)** | 136 (133-139) | 136 (133-138) | .4 |
| **Mean arterial pressure (mmHg) (n=657)** | 92 (82-113) | 90 (80-98) | .9 |
| **Ascites (n=647)** | 233 (46) | 70 (51) | .3 |
| **Hepatic encephalopathy (n=627)** | 105 (21) | 35 (26) | .2 |
| **Hepatocellular carcinoma** | 32 (6) | 9 (6) | .9 |
| **Number of discharge medications (n=448)** | 7 (5-10) | 9 (6-12) | .001 |
| **Substance abuse** | 97 (18) | 16 (11) | .04 |
| **Length of stay (days)** | 4 (2-6) | 4 (3-7) | .15 |
| **Recommended follow-up (GI)** | 225 (42) | 33 (37) | .2 |
| **Followed up in GI clinic** | 228 (43) | 54 (37) | .3 |
| ***Followed in GI clinic within 2 weeks (n=564)** | 29 (7) | 8 (6) | .8 |
| **Interval to GI clinic follow up (days) (n=282)** | 72 (26 – 195)  179±284 | 40 (19 – 68)  99±221 | .05 |
| **30-day mortality (n=405)**  **90-day mortality (n=375)** | 10 (2)  25 (6) | 9 (7)  23 (20) | .01  <.001 |

Abbreviation: INR, International Normalized Ratio, NACSELD-ACLF, North American Consortium for the Study of End-stage Liver Disease-Acute on Chronic Liver Failure

*In patients with at least 2 weeks of follow up post discharge

**Table F.** Comparison of selected characteristics and outcomes of hospitalization in 679 patients discharged alive based on length of stay corresponding to lowest (<2 days) and highest (>10 days) deciles for length of stay. Data are reported as number (percent) or median value (interquartile range).

| **Clinical factor (number of patients if there is missing data)** | **Less than 2 days**  **(n=68)** | **2 to 10 days**  **(n=529)** | **More than 10 days**  **(n=82)** | **P value** |
| --- | --- | --- | --- | --- |
| **Age** | 55 (50 – 59) | 55 (49 – 61) | 56 (51 – 64) | .12 |
| **Gender male** | 45 (66) | 361 (68) | 53 (64) | .8 |
| **Charlson Comorbidity Index** | 3 (3 – 4) | 3 (3 – 5) | 4 (3 – 5) | .04 |
| **Study center**  **Boston Medical Center**  **Ben Taub Hospital**  **Eskenazi Health Hospital**  **John Stroger Hospital** | 24 (35)  16 (23)  14 (21)  14 (21) | 120 (23)  139 (26)  87 (17)  183 (35) | 14 (16)  19 (22)  22 (26)  27 (33) | .13 |
| **Medical Insurance (n=671)**  **Medicaid**  **Medicare**  **Commercial**  **County**  **Jail**  **No insurance**  **Other** | 29 (43)  9 (13)  2 (3)  11 (16)  1 (1.5)  13 (19)  2 (3) | 225 (43)  76 (15)  24 (5)  89 (17)  6 (1)  91 (17)  12 (2) | 33 (41)  17 (20)  5 (6)  10 (12)  1 (1)  14 (17)  1 (1) | .9 |
| **Primary liver related diagnosis**  **Ascites**  **Hepatic encephalopathy**  **Gastrointestinal bleeding** | 30 (44)  19 (28)  3 (4) | 186 (35)  92 (17)  98 (19) | 24 (29)  19 (23)  9 (11) | .06  .6  .4 |
| **Infection on admission** | 7 (10) | 82 (15) | 34 (42) | <.001 |
| **Child Pugh score (n=397)** | 8 (7 – 10) | 8 (7 – 10) | 9 (8 – 11) | .048 |
| **Child Pugh class (n=563)**  **A**  **B**  **C** | 11 (18)  28 (45)  23 (37) | 68(16)  237 (55)  128 (30) | 6 (9)  33 (49)  39 (43) | .2 |
| **NACSELD-ACLF** | None | 3 (0.6) | 7 (9) | <.001 |
| ***Model for End-stage Liver Disease (n=583)** | 14 (12 – 18) | 15 (12 – 19) | 21 (16 – 27) | <.001 |
| **Intensive care on admission (n=665)** | 2 (3) | 62 (12) | 21 (25) | <.001 |
| **30-day all-cause readmission** | 12 (18) | 111 (21) | 22 (26) | .16 |
| **30-day liver-related readmission** | 9 (13) | 66 (12) | 13 (16) | .6 |
| **30-day mortality (n=572)**  **90-day mortality (n=511)** | none  1 (2) | 16 (4)  42 (10) | 3 (4)  5 (9) | .16  .2 |

Abbreviation: NACSELD-ACLF, North American Consortium for the Study of End-stage Liver Disease-Acute on Chronic Liver Failure

Factors not associated with differences in length of stay categories and not shown include; Race, etiology of liver disease, the presence of hepatocellular carcinoma, albumin, admission route, liver related indications of alcoholic hepatitis, acute kidney injury or liver malignancy, and history of tobacco, alcohol or substance use.

**Table G.** The predictors of length of stay > 10 days (the highest decile) in patients surviving the hospitalization on simple and multiple logistic regression.

|  | **Simple logistic regression** | | **Multiple logistic regression** | |
| --- | --- | --- | --- | --- |
| **Clinical variable** | **Odds Ratio (95%CI)** | **P value** | **Odds Ratio (95%CI)** | **P value** |
| **Model for End- stage Liver Disease** | 1.14 (1.1 - 1.18) | <.001 | 1.13 (1.09 – 1.18) | <.001 |
| **Infection on admission** | 4 (2.5 – 6.6) | <.001 | 4.4 (2.4 – 8) | <.001 |
| **NACSELD-ACLF** | 18.5 (4.7 – 73) | <.001 | 9.3 (2 – 43.4) | .004 |
| **Intensive care on admission** | 2.7 (1.6 – 4.8) | <.001 | 2.6 (1.3 – 5.3) | .008 |
| **Child Class C** | 1.7 (1.08 – 2.4) | .04 |  |  |
| **Study Site (relative to Ben Taub General Hospital)**  **Boston University Medical Center**  **Eskenazi Health**  **John Stroger Hospital** | 0.8 (0.4 – 1.6)  1.8 (0.9 – 3.4)  1.3 (0.7 – 2.3) | .5  .09  .7 |  |  |
| **Charlson Comorbidity Index** | 1.1 (0.99 – 1.3) | .07 |  |  |
| **Medical insurance (relative to commercial or Medicare) Medicaid, county or no insurance** | 0.6 (0.35 – 1.05) | .07 |  |  |

Abbreviation: NACSELD-ACLF, North American Consortium for the Study of End-stage Liver Disease-Acute on Chronic Liver Failure

Factors not associated with prolonged length of stay included: age, sex, race, etiology of liver disease, sodium or albumin, liver related indication for hospitalization, and discharge disposition.

The final model was informed by post-estimation analysis with a c-statistic of 0.82 (95% CI 0.77 – 0.87).

**Table H.** The performance of the models predicting hospital mortality and 30-day readmission, when applied to the individual participating centers cohort. Data reported includes c-statistic (95% confidence interval) and p-value.

| **Model endpoint** | **Overall cohort** | **Ben Taub General Hospital** | **Boston Medical Center** | **Eskenazi Health Hospital** | **John Stroger Hospital** |
| --- | --- | --- | --- | --- | --- |
| **Mortality** | 0.88 (0.84 – 0.93)  p < .001 | 0.9 (0.83 – 0.98)  p <.001 | 0.94 (0.88 – 1)  p <.001 | 0.82 (0.72 – 0.91)  p <.001 | 0.91 (0.79 – 0.99)  p <.001 |
| **30-day all-cause readmission** | 0.68 (0.63 – 0.73)  p < .001 | 0.7 (0.6 – 0.79)  p = .035 | 0.73 (0.59 – 0.86)  p = .002 | 0.67 (0.53 – 0.8)  p = .007 | 0.61 (0.52 – 0.7)  p = .09 |
| **Length of stay>10 day** | 0.82 (0.77 – 0.87)  P<.001 | 0.88 (0.78 – 0.98)  p<.001 | 0.86 (0.75 – 0.97)  p.= 002 | 0.82 (0.72 – 0.92)  p<.001 | 0.75 (0.64 – 0.87)  p<.001 |

**Table I.** The results of 1,000 bootstrapping samples, performed to validate the association of model components with the outcomes of mortality and 30-day readmission using samples with even proportions across sites and random samples across sites

Sampling with even proportion across sites – includes site in the model

| Variable | Percent of time significant in hospital mortality multivariate model (p<.05) | Percent of time significant in hospital mortality multivariate model (p<.10) |
| --- | --- | --- |
| MELD | 100% | 100% |
| NACSELD ACLF | 100% | 100% |
| Sodium | 100% | 100% |
| Hepatocellular carcinoma | 100% | 100% |
| White blood cell count | 98% | 99% |
| Site | 0% | 4% |

Random sampling – can have uneven proportions across sites – includes site in the model

| Variable | Percent of time significant in hospital mortality multivariate model (p<.05) | Percent of time significant in hospital mortality multivariate model (p<.10) |
| --- | --- | --- |
| MELD | 100% | 100% |
| NACSELD ACLF | 100% | 100% |
| Sodium | 99% | 100% |
| Hepatocellular carcinoma | 100% | 100% |
| White blood cell count | 100% | 100% |
| Child Class C | 0% | 1% |
| Site | 0% | 4% |

Models included MELD, intensive carte on admission, hepatocellular carcinoma, infection on admission, white blood cell count, race, serum sodium, mean arterial pressure, child class C, NACSELD-ACLF, and study site. Other variables were not included after bivariate analyses.

Sampling with even proportion across sites – includes site in the model

| Variable | Percent of time significant in 30-day readmission multivariate model (p<.05) | Percent of time significant in 30-day readmission multivariate model (p<.10) |
| --- | --- | --- |
| MELD | 100% | 100% |
| Number of medications on discharge | 97% | 100% |
| Gastrointestinal bleeding as the primary liver diagnosis | 70% | 100% |
| Charlson Comorbidity Index > 4 | 39% | 100% |

Random sampling – can have uneven proportions across sites – includes site in the model

| Variable | Percent of time significant in 30-day readmission multivariate model (p<.05) | Percent of time significant in 30-day readmission multivariate model (p<.10) |
| --- | --- | --- |
| MELD | 100% | 100% |
| Number of medications on discharge | 97% | 100% |
| Gastrointestinal bleeding as the primary liver diagnosis | 88% | 100% |
| Charlson Comorbidity Index > 4 | 38% | 99% |

Models included MELD, Child Class C, gastrointestinal bleeding as the primary liver diagnosis, number of medications on discharge, Charlson Comorbidity Index (>4), and site. Other variables were not included after bivariate analyses.

**Table J.** Non-hepatic comorbid conditions comprising the Charlson Comorbidity Index compared in White, Black, and Hispanic patients. Data are reported as number (percentage).

| **Comorbidity** | **White**  **(n=312)** | **Black**  **(n=182)** | **Hispanic**  **(n=164)** | **P value** |
| --- | --- | --- | --- | --- |
| **Diabetes mellitus without complications** | 59 (19) | 29 (16) | 49 (30) | .004 |
| **Diabetes mellitus with complications** | 9 (3) | 16 (9) | 7 (4) | .01 |
| **Malignancy** | 31 (10) | 33 (18) | 11 (7) | .002 |
| **Metastatic malignancy** | 9 (3) | 13 (7) | 3 (2) | .04 |
| **Chronic pulmonary disease** | 37 (12) | 16 (9) | 7 (4) | .01 |
| **Congestive heart failure** | 16 (5) | 29 (16) | 7 (4) | <.001 |
| **Peripheral vascular disease** | None | 4 (2) | None | .005 |
| **Cerebrovascular disease** | 5 (1.6) | 13 (7) | 2 (1.2) | .001 |
| **Acquired Immunodeficiency Syndrome** | 3 (1) | 7 (4) | 3 (2) | .046 |
| **Renal disease** | 22 (7) | 18 (10) | 16 (10) | .3 |
| **Myocardial infarction** | 12 (4) | 7 (4) | 4 (2.4) | .6 |
| **Peptic ulcer disease** | 19 (6) | 9 (5) | 5 (3) | .4 |
| **Hemiplegia/paraplegia** | None | 1 (0.6) | None | .3 |
| **Dementia** | 1 (0.3) | 1 (0.6) | 1 (0.6) | .9 |
| **Rheumatologic disease** | None | 1 (0.6) | 1 (0.6) | .4 |

**Table K.** The predictors of transplant free survival in patients surviving the index hospitalization using multivariate Cox Proportional Hazard Regression analysis.

| **Clinical variable** | **Hazard Ratio (95%CI)** | **P value** |
| --- | --- | --- |
| **Model for End- stage Liver Disease** | 1.05 (1.036 – 1.079) | .002 |
| **Charlson Comorbidity Index > 4** | 2.6 (1.6 – 4.3) | <.001 |
| **Hepatocellular carcinoma** | 2.1 (1.04 – 4.1) | .04 |
